# *MAPT* rs242557 variant is associated with hippocampus tau uptake on ^18^F-AV-1451 PET in non-demented elders

**DOI:** 10.18632/aging.101783

**Published:** 2019-01-31

**Authors:** Xue-Ning Shen, Dan Miao, Jie-Qiong Li, Chen-Chen Tan, Xi-Peng Cao, Lan Tan, Jin-Tai Yu

**Affiliations:** ^1^Department of Neurology and Institute of Neurology, Huashan Hospital, Shanghai Medical College, Fudan University, Shanghai, China; ^2^Department of Neurology, Qingdao Municipal Hospital, Qingdao University, Qingdao, China; ^3^Clinical Research Center, Qingdao Municipal Hospital, Qingdao University, Qingdao, China; ^4^Alzheimer’s Disease Neuroimaging Initiative*

**Keywords:** tauopathy, *MAPT*, hippocampus, ^18^F-AV-1451 PET, non-demented, Alzheimer’s disease

## Abstract

The microtubule-associated protein tau gene (*MAPT*) rs242557 variant is associated with multiple tauopathies and dementia. This study investigated whether it was correlated with brain tau-PET uptake in non-demented elders. Ninety non-demented elders were identified from the Alzheimer's Disease Neuroimaging Initiative cohort. We compared standardized uptake value ratios (SUVRs) of tau-PET tracer ^18^F-AV-1451 between rs242557 variant carriers and non-carriers in 25 regions of interest (ROIs). The minor allele A was associated with increased hippocampus ^18^F-AV-1451 uptake in non-demented elders (left: β = 0.111, Bonferroni corrected p = 0.035; right: β = 0.103, Bonferroni corrected p = 0.031). Aβ-positive participants (left: β = 0.206, Bonferroni corrected p = 0.029; right: β = 0.198, Bonferroni corrected p = 0.035) and *APOE ε*4 non-carriers (left: β = 0.140, Bonferroni corrected p = 0.006; right: β = 0.134, Bonferroni corrected p = 0.004) exhibited approximately the same findings in hippocampus. Considering no obvious associations in other regions, we confirmed the significant correlation of *MAPT* rs242557 risk variant with increased hippocampus tau deposition in non-demented elders. With higher magnitude signals in the hippocampus that is more likely to be uniquely affected in AD, the tau PET ligand ^18^F-AV-1451 seemed to possess a specific binding property for AD-like tau pathology.

## INTRODUCTION

Variations in the microtubule-associated protein tau gene (*MAPT*) which encodes tau protein for microtubule stability and signal transduction, are well documented to be involved in typical tauopathies and dementia [1]. The H1 haplotype has been reported to be a primary factor for the associations of *MAPT* with Alzheimer’s disease (AD), progressive supranuclear palsy (PSP), corticobasal degeneration (CBD) and pathological tau aggregates in cognitively normal elderly [1–3]. Tagging the six-locus sub-haplotype H1c that was predominantly linked to these pathologies, single nucleotide polymorphism (SNP) rs242557 located in a regulatory region interfering with *MAPT* expression [4, 5]. Strong associations have been found of the locus rs242557 with plasma and cerebrospinal fluid (CSF) tau levels [6, 7], but there is a lack of research on the possible correlation between neuroimaging of pathological tau deposits and rs242557 variant. The advent of ^18^F-AV-1451 tracer, a ligand of tau positron emission tomography (PET), has given genetic research the privilege of accurate visualizing the focal distribution and regional propagation of tau pathology in living human brains [8]. Previous studies have demonstrated increased temporal ^18^F-AV-1451 retentions in symptomatic individuals harboring *MAPT* mutations, including R406W and V337M [9, 10]. Researchers have also explored the distinct patterns of ^18^F-AV-1451 binding in relation to clinical variants of AD and other tauopathy [11, 12]. However, it remains insufficient using tau PET to map whether the distribution of brain tau deposition was correlated with *MAPT* rs242557 variant. To test this, we compared the ^18^F-AV-1451 binding between rs242557 variant carriers and non-carriers in a cohort of non-demented elders.

*Data used in preparation of this article were obtained from the Alzheimer’s Disease Neuroimaging Initiative (ADNI) database (adni.loni.usc.edu). As such, the investigators within the ADNI contributed to the design and implementation of ADNI and/or provided data but did not participate in analysis or writing of this report. A complete listing of ADNI investigators can be found at: http://adni.loni.usc.edu/wp-content/uploads/how_to_apply/ADNI_Acknowledgement_List.pdf.

## RESULTS

### Participants

A total of 90 non-demented elders (mean age: 71.39 ± 6.45 years, 62.2% women and mean MMSE score: 28.36 ± 2.02) were enrolled in the study to evaluate the correlation between *MAPT* rs242557 variant and tau-PET ^18^F-AV-1451 uptake in the brain. Demographic characteristics and clinical features of the cohort and subgroups were listed in Table 1. This non-demented cohort was composed of 41 cognitively healthy controls and 49 individuals with mild cognitive impairment (MCI). As expected, the MCI individuals showed worse performance on neuropsychological tests than the normal controls (p < 0.005). We did not find any differences between this two groups in age, gender proportion, years of education and *APOE ε*4 carriage (p > 0.05). While in the subgroup analyses, the Aβ-positive group in comparison to the Aβ-negative group was older (73.03 ± 7.01 vs. 69.91 ± 5.48 years, p < 0.05), presenting with a larger proportion of *APOE ε*4 carriage (45.7% vs. 20.7%, p < 0.05) and worse MMSE scores (27.91 ± 1.84 vs. 28.63 ± 2.10, p < 0.05). There were no significant differences in demographic characteristics and clinical features between the *APOE ε*4 carriers and the non-carriers (p > 0.05).

**Table 1:** Demographic and clinical characteristics of the studying cohort

	Non-demented elders	MCI	CN	Aβ-positive participants	Aβ-negative participants	*APOE ε* 4 carriers	*APOE ε* 4 non-carriers
n	90	49	41	35	53	29	61
Age (years)	71.39 ± 6.45	70.60 ± 6.84	72.34 ± 6.00	73.03 ± 7.01	69.91 ± 5.48 ^b^	70.13 ± 6.89	71.99 ± 6.14
Gender (Male/Female)	34/56	33/16	23/18	14/21	20/33	10/19	24/37
Education (years)	16.39 ± 2.73	16.06 ± 3.14	16.78 ± 2.16	16.40 ± 2.75	16.40 ± 2.77	16.38 ± 3.02	16.39 ± 2.58
Diagnosis (MCI/CN)	49/41	—	—	22/13	26/27	18/11	31/30
Genotype (GG/AG/AA)	44/31/15	25/17/7	19/14/8	16/15/4	28/15/10	13/11/5	31/20/10
*APOE ε*4 carriage (%)	32.2	36.7	26.8	45.7	20.8	—	—
Neuropsychological tests						
MMSE	28.36 ± 2.02	27.77 ± 2.32	29.05 ± 1.36 ^a^	27.91 ± 1.84	28.63 ± 2.10 ^b^	27.93 ± 2.18	28.57 ± 1.90
CDR-SB	0.81 ± 1.34	1.39 ± 1.57	0.09 ± 0.30 ^a^	1.21 ± 1.72	0.58 ± 0.97	1.05 ± 1.68	0.69 ± 1.11
ADAS-cog11	7.19 ± 4.08	8.79 ± 4.53	5.32 ± 2.50 ^a^	8.09 ± 4.70	6.52 ± 3.39	7.34 ± 4.59	7.12 ± 3.81
ADAS-cog13	11.36 ± 6.41	14.21 ± 6.87	8.10 ± 3.94 ^a^	12.66 ± 7.34	10.37 ± 5.62	11.79 ± 7.30	11.15 ± 6.03

Data are presented as mean ± SD; differences in characteristics between sub-groups were assessed using Chi-square test or Wilcoxon rank test.

Note: Two subjects lack of CSF and PET Aβ data were excluded in the analyses grouped by presence or absence of abnormal Aβ deposition.

Aβ = amyloid-beta; APOE = Apolipoprotein E; MCI = mild cognitive impairment; CN = control.

^a^MCI versus CN, p < 0.05.

### Correlations in the whole cohort

**Figure 1:**
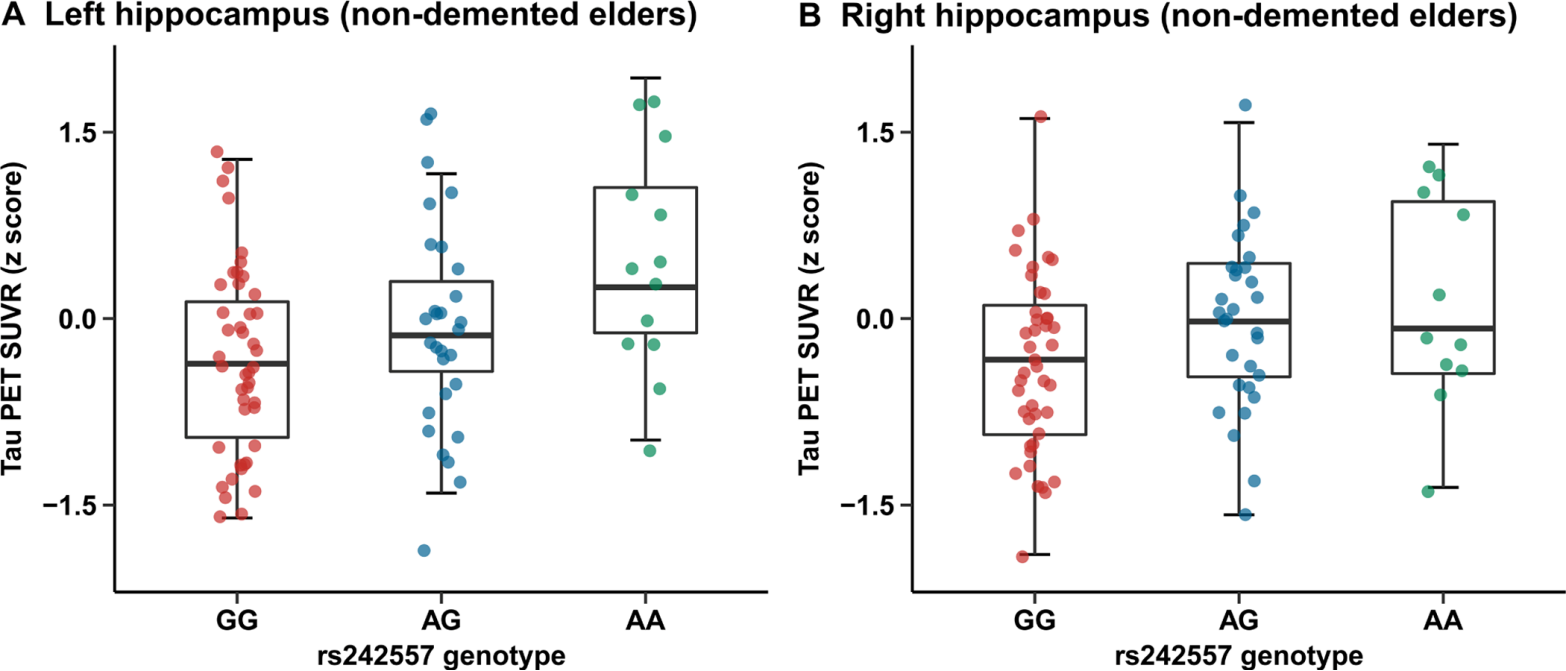
**Quantitative comparisons for adjusted correlations of hippocampus tau PET SUVR levels with rs242557 variant in the non-demented cohort.** (**A**) Left hippocampus tau PET SUVR for each subject in the non-demented elders is plotted separated by rs242557 genotype (GG, AG and AA; adjusted β = 0.111, p = 0.001, Bonferroni corrected p = 0.035). (**B**) Right hippocampus tau PET SUVR for each subject in the non-demented elders is plotted separated by rs242557 genotype (adjusted β = 0.103, p = 0.001, Bonferroni corrected p = 0.031).

Quantitative assessments showed an elevated hippocampus tau-PET SUVR level in subjects carrying rs242557 risk variant (MAF: A=0.4024) compared to the non-carriers in the nondemented cohort (Figure 1A, 1B)). The nonparametric Kruskal-Wallis test suggested that there were rs242557 variant group differences in several brain regions of interest (ROIs) (left/right hippocampus, left entorhinal cortex, left/right parahippocampus, left/right superior temporal cortex, left/right inferior temporal cortex, right lateral occipital cortex, right inferior parietal cortex and left superior frontal cortex, p < 0.05; see Supplementary Table 2). However, the linear regression analyses illustrated that there remained only significant correlations between *MAPT* rs242557 risk variant and elevated hippocampus uptake of ^18^F-AV-1451, whether in the model unadjusted (left: β = 0.128, p < 0.001, Bonferroni corrected p = 0.006 and right: β = 0.120, p < 0.001, Bonferroni corrected p = 0.004) and adjusted (left: β = 0.111, p = 0.001, Bonferroni corrected p = 0.035 and right: β = 0.103, p = 0.001, Bonferroni corrected p = 0.031) for age, gender, education, *APOE* *ε*4 carriage and diagnosis. Analyses of other brain ROIs did not show any significant results (Supplementary Table 1). In addition, we did not observe any group differences among genotypes or statistically significant associations of CSF t-tau and p-tau (Supplementary Tables 1–3).

### Correlations in subgroups categorized by diagnosis

Tau PET signal ^18^F-AV-1451 were statistically different among the 3 genotypes in the left/right hippocampus, left entorhinal cortex, right caudate and left/right superior temporal cortex of the MCI group (p < 0.05) and left parahippocampus of the control group (p < 0.05) (Supplementary Table 2). While in the unadjusted linear regression model, only the left/right hippocampus of the MCI group presented a correlation with higher ^18^F-AV-1451 retention (left: β = 0.164, p = 0.002, Bonferroni corrected p = 0.049 and right: β = 0.163, p = 0.001, Bonferroni corrected p = 0.018), which failed to reduplicate in further analysis adjusted for age, gender, education and *APOE* *ε*4 carriage (left: β = 0.131, p = 0.004, Bonferroni corrected p = 0.103 and right: β = 0.133, p = 0.002, Bonferroni corrected p = 0.051) (Supplementary Table 3).

### Correlations in subgroups categorized by Aβ pathology

**Figure 2:**
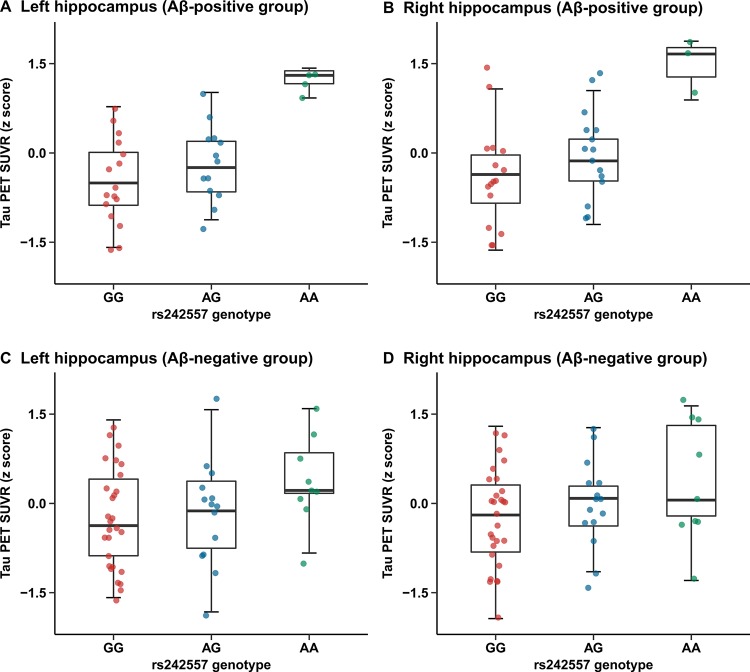
**Quantitative comparisons for adjusted correlations of hippocampus tau PET SUVR levels with rs242557 variant in the subgroups categorized by Aβ pathology.** (**A**) Left hippocampus tau PET SUVR for each subject in the Aβ-positive group is plotted separated by rs242557 genotype (adjusted β = 0.206, p = 0.001, Bonferroni corrected p = 0.029). (**B**) Right hippocampus tau PET SUVR for each subject in the Aβ-positive group is plotted separated by rs242557 genotype (adjusted β = 0.198, p = 0.001, Bonferroni corrected p = 0.035). (**C**) Left hippocampus tau PET SUVR for each subject in the Aβ-negative group is plotted separated by rs242557 genotype (adjusted β = 0.083, p = 0.035, Bonferroni corrected p = 0.878). (**D**) Right hippocampus tau PET SUVR for each subject in the Aβ-negative group is plotted separated by rs242557 genotype (adjusted β = 0.075, p = 0.037, Bonferroni corrected p = 0.930).

Tau-PET SUVRs were greater in several brain regions of subjects carrying the rs242557 allele A than those of non-carriers (left/right hippocampus, left entorhinal cortex, right parahippocampus, right caudate, right superior temporal cortex of the Aβ-positive group and left entorhinal cortex, left parahippocampus, left superior temporal cortex of the Aβ-negative group, p < 0.05) (Supplementary Table 2). In the Aβ-positive group, we observed evidently higher hippocampus ^18^F-AV-1451 retention in rs242557 allele A carriers compared with the non-carriers (unadjusted model: left: β = 0.214, p = 0.001, Bonferroni corrected p = 0.036 and right: β = 0.211, p < 0.001, Bonferroni corrected p = 0.011; adjusted model: left: β = 0.206, p = 0.001, Bonferroni corrected p = 0.029 and right: β = 0.198, p = 0.001, Bonferroni corrected p = 0.035; Figure 2A, 2B), yet this relevance presented unsubstantiated in the Aβ-negative group (unadjusted model: left: β = 0.091, p = 0.014, Bonferroni corrected p = 0.355 and right: β = 0.083, p = 0.015, Bonferroni corrected p = 0.365; adjusted model: left: β = 0.083, p = 0.035, Bonferroni corrected p = 0.878 and right: β = 0.075, p = 0.037, Bonferroni corrected p = 0.930; Figure 2C, 2D).

### Correlations in subgroups categorized by *APOE ε*4 carriage

**Figure 3:**
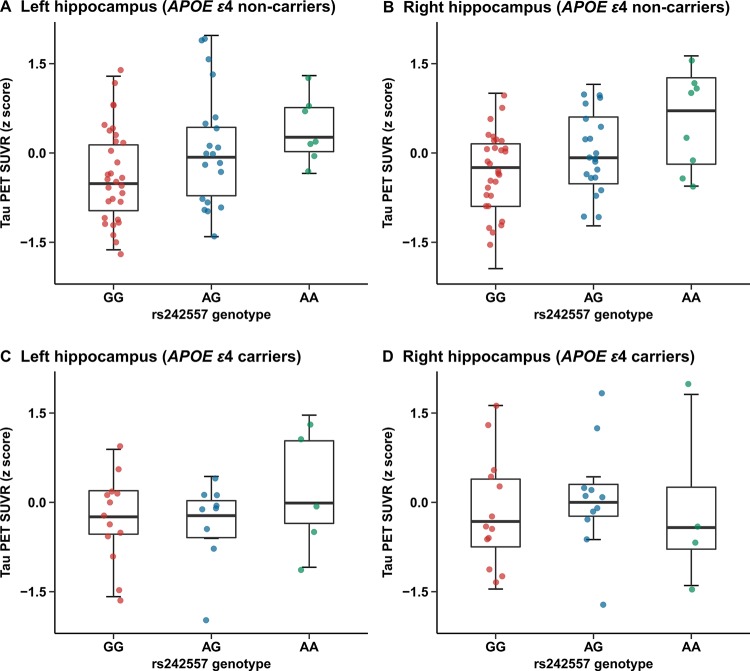
**Quantitative comparisons for adjusted correlations of hippocampus tau PET SUVR levels with rs242557 variant in the subgroups categorized by *APOE ε*4 carriage.** (**A**) Left hippocampus tau PET SUVR for each subject in the *APOE ε*4 non-carriers is plotted separated by rs242557 genotype (adjusted β = 0.140, p < 0.001, Bonferroni corrected p = 0.006). (**B**) Right hippocampus tau PET SUVR for each subject in the *APOE ε*4 non-carriers is plotted separated by rs242557 genotype (adjusted β = 0.134, p < 0.001, Bonferroni corrected p = 0.004). (**C**) Left hippocampus tau PET SUVR for each subject in the *APOE ε*4 carriers is plotted separated by rs242557 genotype (adjusted β = 0.040, p = 0.536). (**D**) Right hippocampus tau PET SUVR for each subject in the *APOE ε*4 carriers is plotted separated by rs242557 genotype (adjusted β = 0.035, p = 0.616).

In the *APOE ε*4 carriers, we did not find significant group differences in any brain ROIs (Supplementary Table 2). While, there were statistical group differences observed in several ROIs of the *APOE ε*4 non-carriers (left/right hippocampus, left entorhinal cortex, left/right parahippocampus, left thalamus, brainstem, left/right superior temporal cortex, left inferior temporal cortex and right superior frontal cortex) (Supplementary Table 2). Further in the more reliable regression analyses, a significant correlation of rs242557 risk variant with increased hippocampus tau-PET ^18^F-AV-1451 uptake has been illustrated in the subgroup of *APOE ε*4 non-carriers (unadjusted model: left: β = 0.148, p < 0.001, Bonferroni corrected p = 0.002 and right: β = 0.144, p < 0.001, Bonferroni corrected p = 0.001; adjusted model: left: β = 0.140, p < 0.001, Bonferroni corrected p = 0.006 and right: β = 0.134, p < 0.001, Bonferroni corrected p = 0.004; Figure 3A, 3B). However, no positive findings were detected in the regression analyses of the *APOE ε*4 carriers (Figure 3C, 3D).

## DISCUSSION

In the present study, we identified a significant correlation between *MAPT* rs242557 risk variant and increased hippocampus uptake of tau-PET tracer ^18^F-AV-1451 in the non-demented elders, especially in those with abnormal Aβ deposition. Subjects harboring rs242557 allele A exhibited higher tau PET SUVR levels than those carrying the allele G, implying that rs242557 served as a functional locus whose minor allele A could promote the expression of tau aggregates in the hippocampus. The widely used tau-PET tracer ^18^F-AV-1451 was developed for binding to the AD-type tau pathology [13]. Recently, a case-control tau-PET study has demonstrated elevated temporal ^18^F-AV-1451 binding in participants with *MAPT* mutations outside exon 10, who were supposed to have 3 repeated (3R-tau) and 4 repeated tau (4R-tau) isoforms mixtures [10]. Since tau aggregates in AD are paired helical filaments (PHF) composed of mixed 3R/4R tau aggregates [14], they therefore supported the notion that ^18^F-AV-1451 has the specific binding capacity for AD-like tau pathology [10]. Our sample of subjects showed higher-magnitude tau-PET ^18^F-AV-1451 signal in the hippocampus rather than other brain ROIs that could be influenced by other non-AD tau pathology. Based on these, it is reasonable to speculate that the ligand ^18^F-AV-1451 may possess a unique binding property for AD-like tau pathology. However, pathophysiologic and longitudinal tau-PET ^18^F-AV-1451 studies would be necessary to investigate whether and how this ligand tracks along with neurodegeneration.

This study added evidence to the correlation of *MAPT* rs242557 variant with tau pathology and helped reconcile some discrepancies concerning the role of rs242557 variant in AD. Previously, Laws *et al* have detected an association between rs242557 minor allele A and elevated cerebrospinal fluid (CSF) total tau (t-tau) levels in a quantitative trait analysis [7]. A genome-wide significant association has also been identified of rs242557 allele A with higher plasma t-tau concentrations [6]. While in a recent meta-analysis of 12 studies, no significant correlations were observed between rs242557 and AD [15]. Authors suggested that the protective impact of rs242557 allele A on risk of AD in the Asian population seemed unreliable and needed further verification eliminating potential influence caused by ethnicity [15]. Our current study limited the ethnicity of included subjects and hence the results were reliable. In addition, it is also illustrated in a subset of amnestic MCI patients with selective medial temporal hypometabolism that absent or low amyloid-PET signal together with CSF β-amyloid (1–42) protein finding did not show expected typical pattern of AD [16]. In another study, patients with R406W mutation exhibited predominant ^18^F-AV-1451 uptake in hippocampus and temporal regions, correlating with glucose hypometabolism and negative amyloid-PET [9]. These provide clues to utilizing the ^18^F-AV-1451 PET for accurately quantifying the regional distribution of pathological tau aggregates [9]. Furthermore, the link between rs242557 variant and significantly increased hippocampus ^18^F-AV-1451 retention was more obvious with the presence of abnormal brain amyloidosis, which may be explained by the possible effect of Aβ pathology as an early pathological change of AD on tau pathology. Besides, we did not find consolidated evidence for correlations in the subgroup analyses categorized by diagnosis. Individuals with MCI did not present a significant correlation of *MAPT* rs242557 variant with pathological tau aggregates in any brain regions. It is speculated that the MCI group might consist of subjects whose cognition decline were caused by other neurodegenerative disorders. Longitudinal studies on large sample size are required for further investigation and verification. In addition, the subgroup analyses by Aβ pathology described the consistent clinical features with the findings reported by Scholl *et al*, that older age and worse cognition performance were associated with elevated tau-PET ^18^F-AV-1451 retention in the medial temporal region [8]. Much effort is needed to further explore the underlying mechanisms. *APOE* *ε*4 is regarded as the most definite genetic factor for AD, and its carriers were found to distribute greater ^18^F-AV-1451 retention in the medial/lateral temporal and parietal cortex than its non-carriers in AD [12]. Our current data identified that rs242557 was merely associated with the^18^F-AV-1451 binding capacity in the hippocampus of *APOE* *ε*4 non-carriers instead of the carriers. We therefore hypothesized that rs242557 risk variant might have an influence on tau aggregates independent of *APOE* *ε*4. Given the small sample size, this statistical association should be interpreted cautiously.

There is still lack of consensus on how rs242557 risk variant of *MAPT* H1c haplotype correlates with increased expression of tau protein in hippocampus. Falling into a highly conservative region, the SNP rs242557 did not illustrate a significant association with messenger RNA (mRNA) expression of *MAPT* [17, 18]. But this conservation was reported to indicate the presence of cis-elements [19], which is pathogenic and will lead to degenerated neurons and subsequent tauopathy [20]. Also, rs242557 minor allele A in the context of H1p promoter of *MAPT* exhibited greater transcription activity than allele G with H1p or H2p promoter variant [4]. Therefore, it could be speculated that the effect of rs242557 on transfer RNA (tRNA) or mRNA splicing and its synergistic or interactive effect with other genetic variants may account for its predominant role [21, 22]. Substantial genetic studies are needed to investigate these possible reasons driven by the locus rs242557 for susceptibility to AD or other tauopathies.

This report has limitations in the small scope of sample size and single tau PET tracer, which may reduce the testing efficiency of whole and stratified analyses to some extent. Given that ethnicity of participants included was defined, our study was likely underpowered to represent this correlation in all individuals who harboring rs242557 risk variant. Besides, there was lack of direct verification in pathologic tau aggregated in the brain. Only multi-centered, clinical and imaging researches with neuropathological verification could provide conclusive evidence for the role of *MAPT* rs242557 variant in AD and other neurodegenerative disorders and the prodromal stages.

In summary, our study explored and has verified the correlation between *MAPT* rs242557 risk variant and tau deposition in hippocampus, especially with the presence of Aβ pathology. The tau-PET tracer ^18^F-AV-1451 showed uniquely higher retention in the hippocampus of *MAPT* rs242557 risk variant carriers who are more likely to develop tau pathology, implying its potential specific binding property for AD-like tau pathology.

## MATERIALS AND METHODS

### Study subjects and ADNI database

We identified and studied subjects with data up to the quality control (QC) criteria from the Alzheimer’s Disease Neuroimaging Initiative (ADNI-1 and ADNI-2) cohort. Detailed and constantly updated information is available on the ADNI database (adni.loni.usc.edu), which was launched as a public-private partnership providing CSF/blood biomarkers, MRI/PET imaging data and genetic information [23]. Established in 2003, the ADNI aimed primarily to explore whether neuroimaging, biological markers, and clinical and neuropsychological assessment can be combined to measure the progression of MCI and early AD. Identification of specific and sensitive biomarkers for disease progression is likely to facilitate the development of promisingly new treatment and help monitoring their effectiveness. Till now, more than 1500 individuals have been recruited from over 50 sites across Canada and the United States, composed of cognitively healthy older participants, patients with MCI and AD.

We searched the database for elderly participants carrying the *MAPT* rs242557 locus. To avoid the potential effect on genotype proportion caused by ethnicity, it is restricted to enroll only the non-Hispanic Caucasian origin. Among these, subjects with either normal cognition or mild cognitive impairment were ultimately included in this present study. In detail, specific inclusion and exclusion criteria are presented in the database (adni.loni.usc.edu/methods/documents). Briefly, the cognitively normal controls were categorized by Mini-Mental State Examination (MMSE) scores ≥ 24 and Clinical Dementia Rating scale (CDR) scores 0 [24, 25]. While, MCI patients were classified with MMSE scores ≥ 24, CDR scores 0.5, objective memory loss that was showed on scores of delayed recall, preservation of activities of daily living, and were not up to criteria for clinical proven AD. Closest imaging and CSF/blood biomarker information in the follow-up period were screened, and those who lack both CSF and PET Aβ data were excluded in the analysis grouped by presence or absence of Aβ pathology.

### Ethic

This study was approved by institutional review boards of all contributing research institutions, and informed consent in writing was acquired from all subjects or authorized agents.

### Genotyping, CSF biomarkers and PET imaging

All samples were genotyped via the Human 610-Quad Bead Chip (Illumina, Inc, San Diego, CA). Data of tau PET imaging assessed by ^18^F-AV-1451, together with CSF t-tau/p-tau and CSF/PET biomarker Aβ were obtained from the constantly updated ADNI database, whose acquisition and processing methods are described in detail. Eligible data on tau PET imaging were corrected for partial volume effects to reduce the effect of atrophy [26]. Standardized uptake value ratios (SUVRs) of tau-PET tracer ^18^F-AV-1451 were compared between *MAPT* rs242557 variant carriers and the non-carriers. To avoid borderline effects, amyloid positive or negative were categorized by the cut-off values which were ± 5% from the original ones [27]. Thus in the present study, subjects with CSF Aβ_42_ levels less than 182.4 pg/ml or summary Aβ-PET SUVR normalized by the whole cerebellum reference region greater than 1.1655 were stratified as have evidence of Aβ pathology (Aβ-positive group), conversely without likely Aβ pathology (Aβ-negative group) [28]. A selective group of brain regions of interest (ROIs) regarded as the most representative regions influenced by tau pathology was investigated separately (hippocampus, entorhinal cortex and parahippocampus for medial temporal cortex; pallidum, caudate and putamen for basal ganglia; brainstem; thalamus; superior/inferior temporal cortex, lateral occipital cortex; superior frontal cortex and inferior parietal cortex) [9, 11, 29, 30].

### Statistical analysis

Tau-PET data value outlying the mean ± 3 SD were regarded as extrema and excluded when analyzed. Differences of demographic characteristics were compared between subgroups (MCI versus control, Aβ-positive group versus Aβ-negative group, apolipoprotein E (*APOE*) *ε*4 carriers versus *APOE ε*4 non-carriers) by Chi-square test or Wilcoxon rank test. Tau-PET SUVRs were set as the dependent variables and standardized to z scores to facilitate comparisons between modalities. The rs242557 variant genotypes (GG, AG and AA) were coded as 0, 1 and 2 to make it accessible for the next phase of analysis. We first explored whether there were any group differences among these 3 genotypes (GG, AG and AA) for tau-PET ^18^F-AV-1451 SUVR in the 25 selected regions. As most of the data were abnormally distributed, comparisons were conducted using the Kruskal-Wallis test. For group differences, a p value of < 0.05 was considered statistically significant. Then for more credible evidence, we utilized the linear regression model to evaluate the associations of tau-PET SUVR levels in brain ROIs with the genetic polymorphism rs242557 (GG, AG and AA). Age, gender, education (years), *APOE* *ε*4 carriage and diagnosis were regarded as confounding variables and included in the multiple linear regression analysis for adjusted associations. A conservative threshold of p < 0.05 after Bonferroni correction for 25 tests was considered to denote significant correlations. All statistical analyses were performed by R 3.23 (www.r-project.org) and PLINK 1.90 software.

## SUPPLEMENTARY MATERIAL

Supplementary Table 1

Supplementary Table 2

Supplementary Table 3
