# Supplementary material for: MAPT rs242557 variant is associated with hippocampus tau uptake on 18F-AV-1451 PET in non-demented elders
Source: Aging (Albany NY). 2019 Jan 31;11(3):874–84. doi: 10.18632/aging.101783 (PMC6382414; doi:10.18632/aging.101783)
Supplement: Supplementary Table 1 [file aging-11-101783-s001.docx]

| **Supplementary Table 1. Quantitative comparisons for unadjusted/adjusted associations of tau PET SUVRs in brain ROIs and CSF t-tau/p-tau with rs242557 variant.** | | | | | | | | | | | | | | | | | | | | | | | | | | | | | | |
| --- | --- | --- | --- | --- | --- | --- | --- | --- | --- | --- | --- | --- | --- | --- | --- | --- | --- | --- | --- | --- | --- | --- | --- | --- | --- | --- | --- | --- | --- | --- |
|  |  | **non-demented elders (n=90)** | | | | |  | **Aβ-positive participants (n=35)** | | | | |  | **Aβ-negative participants (n=53)** | | | | |  | ***APOE* ε4 carriers (n=29)** | | | | |  | ***APOE* ε4 non-carriers (n=61)** | | | | |
|  |  | **unadjusted** | |  | **adjusted** | |  | **unadjusted** | |  | **adjusted** | |  | **unadjusted** | |  | **adjusted** | |  | **unadjusted** | |  | **adjusted** | |  | **unadjusted** | |  | **adjusted** | |
|  |  | **β** | **p** |  | **β** | **p** |  | **β** | **p** |  | **β** | **p** |  | **β** | **p** |  | **β** | **p** |  | **β** | **p** |  | **β** | **p** |  | **β** | **p** |  | **β** | **p** |
| CSF t-tau |  | 2.160 | 0.758 |  | -1.637 | 0.816 |  | 5.156 | 0.728 |  | 3.309 | 0.808 |  | -0.387 | 0.949 |  | -1.003 | 0.872 |  | 1.723 | 0.907 |  | 0.302 | 0.983 |  | 2.292 | 0.777 |  | -1.727 | 0.832 |
| CSF p-tau |  | -3.598 | 0.433 |  | -5.207 | 0.276 |  | -5.65 | 0.603 |  | -7.193 | 0.520 |  | -3.190 | 0.267 |  | -2.614 | 0.392 |  | -9.724 | 0.351 |  | -10.170 | 0.343 |  | -1.103 | 0.827 |  | -2.443 | 0.635 |
| Left hippocampus |  | 0.128 | **0.000*** |  | 0.111 | **0.001*** |  | 0.214 | **0.001*** |  | 0.206 | **0.001*** |  | 0.091 | 0.014 |  | 0.083 | 0.035 |  | 0.077 | 0.295 |  | 0.040 | 0.536 |  | 0.148 | **0.000*** |  | 0.140 | **0.000*** |
| Right hippocampus |  | 0.120 | **0.000*** |  | 0.103 | **0.001*** |  | 0.211 | **0.000*** |  | 0.198 | **0.001*** |  | 0.083 | 0.015 |  | 0.075 | 0.037 |  | 0.063 | 0.342 |  | 0.035 | 0.616 |  | 0.144 | **0.000*** |  | 0.134 | **0.000*** |
| Left entorhinal |  | 0.213 | 0.002 |  | 0.189 | 0.006 |  | 0.330 | 0.006 |  | 0.314 | 0.015 |  | 0.164 | 0.042 |  | 0.151 | 0.075 |  | 0.174 | 0.147 |  | 0.154 | 0.176 |  | 0.226 | 0.008 |  | 0.203 | 0.021 |
| Right entorhinal |  | 0.110 | 0.170 |  | 0.090 | 0.248 |  | 0.012 | 0.925 |  | 0.022 | 0.875 |  | 0.152 | 0.111 |  | 0.147 | 0.132 |  | 0.066 | 0.692 |  | 0.014 | 0.921 |  | 0.122 | 0.167 |  | 0.108 | 0.232 |
| Left parahippocampus |  | 0.141 | 0.007 |  | 0.117 | 0.022 |  | 0.220 | 0.059 |  | 0.179 | 0.146 |  | 0.091 | 0.005 |  | 0.100 | 0.004 |  | 0.093 | 0.350 |  | 0.027 | 0.783 |  | 0.158 | 0.009 |  | 0.149 | 0.018 |
| Right parahippocampus |  | 0.122 | 0.027 |  | 0.099 | 0.071 |  | 0.158 | 0.210 |  | 0.100 | 0.458 |  | 0.088 | 0.023 |  | 0.099 | 0.017 |  | 0.028 | 0.751 |  | -0.021 | 0.825 |  | 0.160 | 0.020 |  | 0.143 | 0.047 |
| Left pallidum |  | 0.024 | 0.661 |  | 0.019 | 0.753 |  | 0.083 | 0.162 |  | 0.073 | 0.237 |  | -0.043 | 0.585 |  | -0.067 | 0.431 |  | 0.087 | 0.432 |  | 0.102 | 0.409 |  | -0.008 | 0.905 |  | -0.026 | 0.696 |
| Right pallidum |  | -0.006 | 0.895 |  | -0.008 | 0.881 |  | 0.049 | 0.388 |  | 0.052 | 0.374 |  | -0.063 | 0.370 |  | -0.082 | 0.282 |  | 0.058 | 0.495 |  | 0.080 | 0.398 |  | -0.036 | 0.558 |  | -0.050 | 0.434 |
| Left caudate |  | 0.017 | 0.655 |  | -0.004 | 0.927 |  | 0.037 | 0.564 |  | 0.028 | 0.659 |  | -0.003 | 0.948 |  | -0.016 | 0.756 |  | -0.011 | 0.867 |  | -0.057 | 0.354 |  | 0.027 | 0.553 |  | 0.006 | 0.900 |
| Right caudate |  | 0.063 | 0.072 |  | 0.048 | 0.191 |  | 0.119 | 0.065 |  | 0.107 | 0.120 |  | 0.035 | 0.421 |  | 0.032 | 0.488 |  | 0.074 | 0.260 |  | 0.047 | 0.444 |  | 0.058 | 0.174 |  | 0.036 | 0.415 |
| Left putamen |  | 0.035 | 0.291 |  | 0.019 | 0.583 |  | 0.085 | 0.197 |  | 0.070 | 0.286 |  | 0.002 | 0.968 |  | -0.007 | 0.867 |  | 0.053 | 0.369 |  | 0.014 | 0.796 |  | 0.025 | 0.540 |  | 0.010 | 0.814 |
| Right putamen |  | 0.032 | 0.369 |  | 0.013 | 0.711 |  | 0.090 | 0.159 |  | 0.073 | 0.246 |  | -0.004 | 0.930 |  | -0.011 | 0.818 |  | 0.051 | 0.453 |  | 0.010 | 0.877 |  | 0.020 | 0.632 |  | 0.005 | 0.902 |
| Left thalamus |  | 0.023 | 0.132 |  | 0.027 | 0.828 |  | 0.035 | 0.202 |  | 0.014 | 0.635 |  | 0.011 | 0.558 |  | -0.010 | 0.679 |  | 0.000 | 0.994 |  | -0.012 | 0.704 |  | 0.033 | 0.102 |  | 0.005 | 0.835 |
| Right thalamus |  | -0.001 | 0.971 |  | 0.004 | 0.259 |  | 0.011 | 0.737 |  | 0.004 | 0.897 |  | -0.010 | 0.679 |  | 0.021 | 0.408 |  | -0.007 | 0.828 |  | 0.001 | 0.977 |  | 0.000 | 0.998 |  | 0.025 | 0.320 |
| Brainstem |  | 0.020 | 0.294 |  | 0.022 | 0.080 |  | 0.009 | 0.788 |  | 0.033 | 0.198 |  | 0.021 | 0.408 |  | 0.013 | 0.500 |  | 0.007 | 0.803 |  | 0.001 | 0.968 |  | 0.024 | 0.319 |  | 0.035 | 0.077 |
| Left superior temporal cortex |  | 0.099 | 0.004 |  | 0.077 | 0.028 |  | 0.170 | 0.038 |  | 0.127 | 0.126 |  | 0.065 | 0.010 |  | 0.065 | 0.016 |  | 0.032 | 0.454 |  | -0.002 | 0.954 |  | 0.131 | 0.005 |  | 0.107 | 0.025 |
| Right superior temporal cortex |  | 0.059 | 0.050 |  | 0.038 | 0.216 |  | 0.096 | 0.169 |  | 0.063 | 0.367 |  | 0.033 | 0.202 |  | 0.030 | 0.285 |  | 0.073 | 0.113 |  | 0.042 | 0.366 |  | 0.051 | 0.193 |  | 0.027 | 0.502 |
| Left inferior temporal cortex |  | 0.119 | 0.074 |  | 0.077 | 0.259 |  | 0.206 | 0.227 |  | 0.127 | 0.476 |  | 0.075 | 0.025 |  | 0.073 | 0.041 |  | 0.019 | 0.838 |  | -0.040 | 0.639 |  | 0.166 | 0.062 |  | 0.119 | 0.192 |
| Right inferior temporal cortex |  | 0.067 | 0.269 |  | 0.022 | 0.714 |  | 0.046 | 0.760 |  | -0.031 | 0.840 |  | 0.067 | 0.043 |  | 0.063 | 0.078 |  | 0.060 | 0.490 |  | -0.009 | 0.908 |  | 0.068 | 0.401 |  | 0.020 | 0.807 |
| Left lateral occipital cortex |  | 0.087 | 0.235 |  | 0.083 | 0.262 |  | 0.068 | 0.722 |  | 0.103 | 0.605 |  | 0.098 | 0.031 |  | 0.108 | 0.026 |  | 0.037 | 0.836 |  | -0.003 | 0.985 |  | 0.106 | 0.128 |  | 0.109 | 0.140 |
| Right lateral occipital cortex |  | 0.133 | 0.047 |  | 0.123 | 0.059 |  | 0.258 | 0.133 |  | 0.302 | 0.061 |  | 0.075 | 0.061 |  | 0.079 | 0.067 |  | 0.246 | 0.179 |  | 0.223 | 0.211 |  | 0.072 | 0.126 |  | 0.065 | 0.194 |
| Left inferior parietal cortex |  | 0.061 | 0.202 |  | 0.029 | 0.553 |  | 0.061 | 0.604 |  | 0.005 | 0.966 |  | 0.056 | 0.067 |  | 0.056 | 0.090 |  | 0.010 | 0.919 |  | -0.091 | 0.321 |  | 0.081 | 0.124 |  | 0.069 | 0.210 |
| Right inferior parietal cortex |  | 0.055 | 0.283 |  | 0.022 | 0.676 |  | 0.058 | 0.655 |  | -0.011 | 0.934 |  | 0.041 | 0.160 |  | 0.048 | 0.132 |  | 0.081 | 0.390 |  | -0.007 | 0.935 |  | 0.039 | 0.527 |  | 0.017 | 0.789 |
| Left superior frontal cortex |  | 0.043 | 0.084 |  | 0.032 | 0.204 |  | 0.016 | 0.762 |  | -0.001 | 0.992 |  | 0.049 | 0.061 |  | 0.050 | 0.074 |  | 0.017 | 0.714 |  | -0.008 | 0.856 |  | 0.054 | 0.073 |  | 0.043 | 0.164 |
| Right superior frontal cortex |  | 0.035 | 0.304 |  | 0.019 | 0.591 |  | -0.025 | 0.762 |  | -0.061 | 0.497 |  | 0.059 | 0.032 |  | 0.062 | 0.035 |  | 0.005 | 0.920 |  | -0.023 | 0.653 |  | 0.047 | 0.291 |  | 0.027 | 0.560 |

Unadjusted and adjusted data (β and p values) are listed in the table.

Note: Two subjects lack of CSF and PET Aβ data were excluded in the analysis grouped by presence or absence of Aβ pathology.

Aβ = amyloid-beta, APOE = Apolipoprotein E.

* Bonferroni corrected p value < 0.05.
