# Supplementary material for: MAPT rs242557 variant is associated with hippocampus tau uptake on 18F-AV-1451 PET in non-demented elders
Source: Aging (Albany NY). 2019 Jan 31;11(3):874–84. doi: 10.18632/aging.101783 (PMC6382414; doi:10.18632/aging.101783)
Supplement: Supplementary Table 2 [file aging-11-101783-s002.docx]

| **Supplementary Table 2. Group differences of genotypes in the non-demented cohort and subgroups.** | | | | | | | | | | | | | | | |
| --- | --- | --- | --- | --- | --- | --- | --- | --- | --- | --- | --- | --- | --- | --- | --- |
|  |  | **non-demented elders (n = 90)** | | | |  | **MCI (n = 49)** | | | |  | **CN (n = 41)** | | | |
|  |  | **GG** | **AG** | **AA** | **p value** |  | **GG** | **AG** | **AA** | **p value** |  | **GG** | **AG** | **AA** | **p value** |
| CSF t-tau |  | 68.600 | 67.300 | 63.400 | 0.953 |  | 73.150 | 68.400 | 78.650 | 0.722 |  | 66.900 | 66.200 | 65.700 | 0.772 |
| CSF p-tau |  | 37.750 | 38.250 | 34.100 | 0.631 |  | 41.000 | 32.700 | 43.150 | 0.531 |  | 37.750 | 37.300 | 31.700 | 0.290 |
| Left hippocampus |  | 1.190 | 1.285 | 1.392 | **0.004** |  | 1.166 | 1.261 | 1.598 | **0.015** |  | 1.193 | 1.310 | 1.425 | 0.172 |
| Right hippocampus |  | 1.165 | 1.246 | 1.432 | **0.009** |  | 1.146 | 1.252 | 1.620 | **0.011** |  | 1.173 | 1.254 | 1.464 | 0.330 |
| Left entorhinal |  | 1.416 | 1.553 | 1.698 | **0.007** |  | 1.416 | 1.677 | 2.253 | **0.047** |  | 1.264 | 1.436 | 1.765 | 0.093 |
| Right entorhinal |  | 1.333 | 1.634 | 1.585 | 0.096 |  | 1.381 | 1.644 | 1.880 | 0.463 |  | 1.263 | 1.463 | 1.656 | 0.103 |
| Left parahippocampus |  | 1.083 | 1.319 | 1.377 | **0.004** |  | 1.128 | 1.324 | 1.492 | 0.061 |  | 1.045 | 1.115 | 1.351 | **0.038** |
| Right parahippocampus |  | 1.136 | 1.283 | 1.401 | **0.013** |  | 1.154 | 1.287 | 1.612 | 0.094 |  | 1.118 | 1.269 | 1.452 | 0.095 |
| Left pallidum |  | 1.912 | 1.884 | 1.826 | 0.932 |  | 1.943 | 1.881 | 1.814 | 0.720 |  | 1.916 | 1.888 | 1.838 | 0.996 |
| Right pallidum |  | 1.848 | 1.745 | 1.855 | 0.422 |  | 1.836 | 1.753 | 1.839 | 0.707 |  | 1.848 | 1.653 | 1.876 | 0.269 |
| Left caudate |  | 1.361 | 1.412 | 1.382 | 0.785 |  | 1.323 | 1.406 | 1.365 | 0.553 |  | 1.395 | 1.381 | 1.423 | 0.995 |
| Right caudate |  | 1.366 | 1.392 | 1.576 | 0.064 |  | 1.306 | 1.301 | 1.600 | **0.023** |  | 1.426 | 1.372 | 1.549 | 0.846 |
| Left putamen |  | 1.447 | 1.449 | 1.467 | 0.593 |  | 1.438 | 1.428 | 1.503 | 0.333 |  | 1.453 | 1.441 | 1.503 | 0.955 |
| Right putamen |  | 1.535 | 1.498 | 1.555 | 0.658 |  | 1.490 | 1.469 | 1.584 | 0.329 |  | 1.592 | 1.504 | 1.625 | 0.989 |
| Left thalamus |  | 0.964 | 0.980 | 0.972 | 0.265 |  | 0.964 | 1.000 | 0.940 | 0.548 |  | 0.976 | 0.962 | 1.014 | 0.463 |
| Right thalamus |  | 1.168 | 1.125 | 1.160 | 0.499 |  | 1.185 | 1.125 | 1.170 | 0.458 |  | 1.167 | 1.133 | 1.155 | 0.953 |
| Brainstem |  | 1.157 | 1.098 | 1.165 | 0.091 |  | 1.163 | 1.061 | 1.189 | 0.102 |  | 1.165 | 1.096 | 1.184 | 0.694 |
| Left superior temporal cortex |  | 1.160 | 1.229 | 1.341 | **0.004** |  | 1.139 | 1.218 | 1.338 | **0.005** |  | 1.191 | 1.230 | 1.355 | 0.386 |
| Right superior temporal cortex |  | 1.200 | 1.192 | 1.362 | **0.046** |  | 1.210 | 1.187 | 1.490 | **0.013** |  | 1.212 | 1.247 | 1.334 | 0.689 |
| Left inferior temporal cortex |  | 1.449 | 1.565 | 1.655 | **0.012** |  | 1.465 | 1.541 | 1.720 | 0.094 |  | 1.449 | 1.548 | 1.597 | 0.115 |
| Right inferior temporal cortex |  | 1.455 | 1.508 | 1.643 | **0.032** |  | 1.462 | 1.501 | 1.892 | 0.068 |  | 1.423 | 1.515 | 1.638 | 0.193 |
| Left lateral occipital cortex |  | 1.344 | 1.366 | 1.441 | 0.164 |  | 1.385 | 1.412 | 1.452 | 0.469 |  | 1.301 | 1.298 | 1.487 | 0.335 |
| Right lateral occipital cortex |  | 1.313 | 1.365 | 1.480 | **0.042** |  | 1.344 | 1.377 | 1.643 | 0.109 |  | 1.289 | 1.301 | 1.537 | 0.174 |
| Left inferior parietal cortex |  | 1.325 | 1.394 | 1.415 | 0.103 |  | 1.410 | 1.373 | 1.440 | 0.284 |  | 1.295 | 1.338 | 1.439 | 0.223 |
| Right inferior parietal cortex |  | 1.316 | 1.368 | 1.493 | **0.036** |  | 1.351 | 1.302 | 1.607 | 0.064 |  | 1.298 | 1.365 | 1.525 | 0.196 |
| Left superior frontal cortex |  | 1.059 | 1.101 | 1.138 | **0.049** |  | 1.076 | 1.108 | 1.161 | 0.120 |  | 1.045 | 1.084 | 1.136 | 0.218 |
| Right superior frontal cortex |  | 1.091 | 1.111 | 1.183 | 0.070 |  | 1.097 | 1.111 | 1.247 | 0.056 |  | 1.097 | 1.100 | 1.154 | 0.402 |

**(continued table)**

|  | **Aβ-positive participants (n = 35)** | | | |  | **Aβ-negative participants (n = 53)** | | | |  | ***APOE ε*4 carriers (n = 29)** | | | |  | ***APOE ε*4 non-carriers (n = 61)** | | | |
| --- | --- | --- | --- | --- | --- | --- | --- | --- | --- | --- | --- | --- | --- | --- | --- | --- | --- | --- | --- |
|  | **GG** | **AG** | **AA** | **p value** |  | **GG** | **AG** | **AA** | **p value** |  | **GG** | **AG** | **AA** | **p value** |  | **GG** | **AG** | **AA** | **p value** |
| CSF t-tau | 101.800 | 66.200 | 101.200 | 0.897 |  | 62.800 | 68.500 | 57.000 | 0.896 |  | 66.200 | 65.000 | 60.200 | 0.856 |  | 69.600 | 73.000 | 74.400 | 0.755 |
| CSF p-tau | 46.800 | 39.200 | 52.500 | 0.653 |  | 30.700 | 37.300 | 28.500 | 0.565 |  | 29.900 | 27.600 | 36.150 | 0.619 |  | 39.400 | 39.800 | 34.100 | 0.618 |
| Left hippocampus | 1.213 | 1.322 | 1.754 | **0.008** |  | 1.154 | 1.191 | 1.258 | 0.068 |  | 1.230 | 1.442 | 1.447 | 0.716 |  | 1.137 | 1.202 | 1.392 | **0.002** |
| Right hippocampus | 1.241 | 1.299 | 1.788 | **0.005** |  | 1.159 | 1.167 | 1.189 | 0.151 |  | 1.138 | 1.336 | 1.450 | 0.677 |  | 1.165 | 1.174 | 1.432 | **0.004** |
| Left entorhinal | 1.596 | 1.782 | 2.432 | **0.015** |  | 1.222 | 1.360 | 1.431 | **0.026** |  | 1.496 | 1.782 | 1.861 | 0.379 |  | 1.266 | 1.509 | 1.698 | **0.017** |
| Right entorhinal | 1.694 | 1.753 | 1.918 | 0.518 |  | 1.243 | 1.367 | 1.414 | 0.098 |  | 1.491 | 1.753 | 1.597 | 0.654 |  | 1.304 | 1.463 | 1.585 | 0.181 |
| Left parahippocampus | 1.266 | 1.496 | 2.021 | 0.071 |  | 1.057 | 1.146 | 1.258 | **0.026** |  | 1.121 | 1.391 | 1.390 | 0.515 |  | 1.073 | 1.229 | 1.377 | **0.006** |
| Right parahippocampus | 1.229 | 1.379 | 1.890 | **0.044** |  | 1.099 | 1.154 | 1.152 | 0.114 |  | 1.234 | 1.379 | 1.449 | 0.586 |  | 1.129 | 1.247 | 1.401 | **0.009** |
| Left pallidum | 1.855 | 1.888 | 1.900 | 0.627 |  | 1.947 | 1.872 | 1.826 | 0.814 |  | 1.916 | 2.116 | 1.676 | 0.097 |  | 1.907 | 1.833 | 1.900 | 0.355 |
| Right pallidum | 1.847 | 1.741 | 1.876 | 0.334 |  | 1.854 | 1.753 | 1.764 | 0.529 |  | 1.770 | 1.999 | 1.655 | 0.667 |  | 1.878 | 1.741 | 1.886 | 0.073 |
| Left caudate | 1.329 | 1.395 | 1.392 | 0.973 |  | 1.387 | 1.418 | 1.382 | 0.969 |  | 1.337 | 1.418 | 1.242 | 0.433 |  | 1.385 | 1.406 | 1.409 | 0.508 |
| Right caudate | 1.370 | 1.240 | 1.769 | **0.035** |  | 1.366 | 1.412 | 1.427 | 0.440 |  | 1.337 | 1.457 | 1.515 | 0.336 |  | 1.382 | 1.301 | 1.576 | 0.080 |
| Left putamen | 1.508 | 1.451 | 1.858 | 0.089 |  | 1.440 | 1.446 | 1.372 | 0.875 |  | 1.422 | 1.611 | 1.419 | 0.801 |  | 1.465 | 1.446 | 1.539 | 0.404 |
| Right putamen | 1.614 | 1.528 | 1.923 | 0.114 |  | 1.508 | 1.455 | 1.422 | 0.966 |  | 1.500 | 1.688 | 1.512 | 0.771 |  | 1.580 | 1.455 | 1.555 | 0.429 |
| Left thalamus | 0.914 | 0.962 | 0.985 | 0.472 |  | 0.970 | 1.000 | 0.972 | 0.740 |  | 0.984 | 1.024 | 0.895 | 0.314 |  | 0.960 | 0.944 | 1.049 | **0.040** |
| Right thalamus | 1.116 | 1.105 | 1.184 | 0.917 |  | 1.177 | 1.126 | 1.160 | 0.317 |  | 1.184 | 1.215 | 1.038 | 0.560 |  | 1.166 | 1.097 | 1.183 | 0.081 |
| Brainstem | 1.176 | 1.061 | 1.245 | 0.101 |  | 1.157 | 1.117 | 1.158 | 0.639 |  | 1.200 | 1.162 | 1.080 | 0.893 |  | 1.151 | 1.061 | 1.203 | **0.025** |
| Left superior temporal cortex | 1.228 | 1.288 | 1.401 | 0.097 |  | 1.153 | 1.211 | 1.315 | **0.026** |  | 1.169 | 1.288 | 1.263 | 0.419 |  | 1.158 | 1.218 | 1.341 | **0.006** |
| Right superior temporal cortex | 1.194 | 1.204 | 1.701 | **0.017** |  | 1.210 | 1.187 | 1.330 | 0.555 |  | 1.234 | 1.295 | 1.426 | 0.584 |  | 1.195 | 1.190 | 1.362 | **0.049** |
| Left inferior temporal cortex | 1.500 | 1.583 | 1.794 | 0.124 |  | 1.423 | 1.541 | 1.489 | 0.071 |  | 1.481 | 1.663 | 1.597 | 0.350 |  | 1.443 | 1.541 | 1.655 | **0.036** |
| Right inferior temporal cortex | 1.491 | 1.515 | 2.184 | 0.050 |  | 1.415 | 1.477 | 1.474 | 0.159 |  | 1.481 | 1.808 | 1.744 | 0.440 |  | 1.443 | 1.477 | 1.643 | 0.061 |
| Left lateral occipital cortex | 1.382 | 1.387 | 1.509 | 0.935 |  | 1.214 | 1.346 | 1.415 | 0.071 |  | 1.414 | 1.793 | 1.356 | 0.699 |  | 1.315 | 1.331 | 1.441 | 0.155 |
| Right lateral occipital cortex | 1.344 | 1.382 | 1.771 | 0.055 |  | 1.297 | 1.352 | 1.450 | 0.178 |  | 1.355 | 1.493 | 1.586 | 0.307 |  | 1.294 | 1.305 | 1.469 | 0.118 |
| Left inferior parietal cortex | 1.351 | 1.599 | 1.490 | 0.500 |  | 1.314 | 1.327 | 1.412 | 0.271 |  | 1.386 | 1.730 | 1.391 | 0.681 |  | 1.322 | 1.327 | 1.415 | 0.167 |
| Right inferior parietal cortex | 1.346 | 1.420 | 1.754 | 0.063 |  | 1.294 | 1.268 | 1.365 | 0.303 |  | 1.354 | 1.488 | 1.574 | 0.352 |  | 1.302 | 1.302 | 1.493 | 0.095 |
| Left superior frontal cortex | 1.059 | 1.173 | 1.217 | 0.437 |  | 1.064 | 1.019 | 1.134 | 0.192 |  | 1.071 | 1.173 | 1.120 | 0.554 |  | 1.048 | 1.087 | 1.138 | 0.076 |
| Right superior frontal cortex | 1.143 | 1.112 | 1.274 | 0.353 |  | 1.087 | 1.111 | 1.150 | 0.196 |  | 1.134 | 1.145 | 1.156 | 0.929 |  | 1.083 | 1.090 | 1.183 | **0.044** |
