# Supplementary material for: MAPT rs242557 variant is associated with hippocampus tau uptake on 18F-AV-1451 PET in non-demented elders
Source: Aging (Albany NY). 2019 Jan 31;11(3):874–84. doi: 10.18632/aging.101783 (PMC6382414; doi:10.18632/aging.101783)
Supplement: Supplementary Table 3 [file aging-11-101783-s003.docx]

| **Supplementary Table 3. Quantitative comparisons for unadjusted/adjusted associations of tau PET SUVRs in brain ROIs and CSF t-tau/p-tau with rs242557 variant in subgroups categorized by diagnosis.** | | | | | | | | | | | | |
| --- | --- | --- | --- | --- | --- | --- | --- | --- | --- | --- | --- | --- |
|  |  | **MCI (n = 49)** | | | | |  | **CN (n = 41)** | | | | |
|  |  | **unadjusted** | |  | **adjusted** | |  | **unadjusted** | |  | **adjusted** | |
|  |  | **β** | **p** |  | **β** | **p** |  | **β** | **p** |  | **β** | **p** |
| CSF t-tau |  | 6.124 | 0.562 |  | 3.170 | 0.755 |  | -1.452 | 0.875 |  | -2.083 | 0.835 |
| CSF p-tau |  | -5.794 | 0.363 |  | -6.386 | 0.338 |  | -1.031 | 0.879 |  | -2.831 | 0.705 |
| Left hippocampus |  | 0.164 | **0.002*** |  | 0.131 | 0.004 |  | 0.094 | 0.036 |  | 0.102 | 0.036 |
| Right hippocampus |  | 0.163 | **0.001*** |  | 0.133 | 0.002 |  | 0.079 | 0.049 |  | 0.093 | 0.035 |
| Left entorhinal |  | 0.280 | 0.015 |  | 0.229 | 0.042 |  | 0.159 | 0.023 |  | 0.170 | 0.028 |
| Right entorhinal |  | 0.135 | 0.316 |  | 0.094 | 0.472 |  | 0.102 | 0.199 |  | 0.090 | 0.285 |
| Left parahippocampus |  | 0.155 | 0.062 |  | 0.103 | 0.190 |  | 0.140 | 0.014 |  | 0.140 | 0.029 |
| Right parahippocampus |  | 0.135 | 0.147 |  | 0.080 | 0.385 |  | 0.123 | 0.012 |  | 0.125 | 0.024 |
| Left pallidum |  | 0.046 | 0.604 |  | 0.024 | 0.800 |  | 0.000 | 0.997 |  | 0.022 | 0.759 |
| Right pallidum |  | 0.023 | 0.761 |  | 0.005 | 0.956 |  | -0.037 | 0.536 |  | -0.023 | 0.730 |
| Left caudate |  | 0.041 | 0.456 |  | 0.017 | 0.758 |  | -0.014 | 0.778 |  | -0.024 | 0.664 |
| Right caudate |  | 0.118 | 0.017 |  | 0.091 | 0.062 |  | 0.000 | 1.000 |  | 0.007 | 0.895 |
| Left putamen |  | 0.062 | 0.188 |  | 0.030 | 0.499 |  | 0.005 | 0.923 |  | 0.010 | 0.848 |
| Right putamen |  | 0.052 | 0.307 |  | 0.014 | 0.769 |  | 0.009 | 0.863 |  | 0.021 | 0.703 |
| Left thalamus |  | 0.026 | 0.190 |  | 0.027 | 0.161 |  | 0.018 | 0.442 |  | 0.033 | 0.172 |
| Right thalamus |  | 0.004 | 0.892 |  | 0.003 | 0.902 |  | -0.006 | 0.851 |  | 0.007 | 0.825 |
| Brainstem |  | 0.025 | 0.349 |  | 0.018 | 0.484 |  | 0.013 | 0.650 |  | 0.033 | 0.255 |
| Left superior temporal cortex |  | 0.085 | 0.032 |  | 0.063 | 0.107 |  | 0.110 | 0.059 |  | 0.096 | 0.140 |
| Right superior temporal cortex |  | 0.087 | 0.070 |  | 0.061 | 0.196 |  | 0.031 | 0.394 |  | 0.028 | 0.497 |
| Left inferior temporal cortex |  | 0.055 | 0.577 |  | -0.004 | 0.970 |  | 0.192 | 0.035 |  | 0.180 | 0.076 |
| Right inferior temporal cortex |  | 0.069 | 0.510 |  | 0.005 | 0.963 |  | 0.077 | 0.158 |  | 0.065 | 0.283 |
| Left lateral occipital cortex |  | 0.102 | 0.443 |  | 0.076 | 0.566 |  | 0.086 | 0.068 |  | 0.091 | 0.082 |
| Right lateral occipital cortex |  | 0.199 | 0.099 |  | 0.174 | 0.128 |  | 0.082 | 0.031 |  | 0.088 | 0.039 |
| Left inferior parietal cortex |  | 0.030 | 0.705 |  | -0.010 | 0.900 |  | 0.100 | 0.064 |  | 0.080 | 0.166 |
| Right inferior parietal cortex |  | 0.045 | 0.611 |  | 0.001 | 0.995 |  | 0.074 | 0.108 |  | 0.064 | 0.208 |
| Left superior frontal cortex |  | 0.050 | 0.210 |  | 0.022 | 0.560 |  | 0.038 | 0.199 |  | 0.056 | 0.069 |
| Right superior frontal cortex |  | 0.035 | 0.562 |  | -0.002 | 0.977 |  | 0.041 | 0.190 |  | 0.056 | 0.102 |

Unadjusted and adjusted data (β and p values) are listed in the table.

MCI, mild cognitive impairment; CN, control.

*Bonferroni corrected p value < 0.05.
